# CD133 (Prominin) Negative Human Neural Stem Cells Are Clonogenic and Tripotent

**DOI:** 10.1371/journal.pone.0005498

**Published:** 2009-05-11

**Authors:** Yirui Sun, Weiqing Kong, Anna Falk, Jin Hu, Liangfu Zhou, Steve Pollard, Austin Smith

**Affiliations:** 1 Wellcome Trust Centre for Stem Cell Research, and Department of Biochemistry, University of Cambridge, Cambridge, United Kingdom; 2 Shanghai No. 6 People's Hospital, Jiao Tong University, Shanghai, People's Republic of China; 3 Huashan Hospital, Fudan University, Shanghai, People's Republic of China; Institut de la Vision, France

## Abstract

**Background:**

CD133 (Prominin) is widely used as a marker for the identification and isolation of neural precursor cells from normal brain or tumor tissue. However, the assumption that CD133 is expressed constitutively in neural precursor cells has not been examined.

**Methodology/Principal Findings:**

In this study, we demonstrate that CD133 and a second marker CD15 are expressed heterogeneously in uniformly undifferentiated human neural stem (NS) cell cultures. After fractionation by flow cytometry, clonogenic tripotent cells are found in populations negative or positive for either marker. We further show that CD133 is down-regulated at the mRNA level in cells lacking CD133 immunoreactivity. Cell cycle profiling reveals that CD133 negative cells largely reside in G1/G0, while CD133 positive cells are predominantly in S, G2, or M phase. A similar pattern is apparent in mouse NS cell lines. Compared to mouse NS cells, however, human NS cell cultures harbour an increased proportion of CD133 negative cells and display a longer doubling time. This may in part reflect a sub-population of slow- or non-cycling cells amongst human NS cells because we find that around 5% of cells do not take up BrdU over a 14-day labelling period. Non-proliferating NS cells remain undifferentiated and at least some of them are capable of re-entry into the cell cycle and subsequent continuous expansion.

**Conclusions:**

The finding that a significant fraction of clonogenic neural stem cells lack the established markers CD133 and CD15, and that some of these cells may be dormant or slow-cycling, has implications for approaches to identify and isolate neural stem cells and brain cancer stem cells. Our data also suggest the possibility that CD133 may be specifically down-regulated during G0/G1, and this should be considered when this marker is used to identify and isolate other tissue and cancer stem cells.

## Introduction

Findings of continuous neurogenesis in the mammal central nervous system (CNS) have raised great interest in neural stem and progenitor cells in both basic and applied neurobiology [1]–[5]. However, interogation of neural stem cells is hampered by the lack of specific markers. Proteins such as Nestin, Musashi, Sox2, and glial fibrillary acidic protein (GFAP) are expressed in neural precursor cells [6]–[9], but they are also expressed by other cell types. More importantly, intracellular markers cannot be used for prospective stem cell isolation, although in mice this problem may be circumvented by creating transgenic reporter animals [10]–[13]. Recent studies have indicated that certain cell surface markers can be used to locate and enrich neural precursor cells. Capela and Temple (2002) isolated proliferative and neurogenic precursor cells from adult mouse subventricular zone (SVZ) by harvesting cells expressing LeX, a carbohydrate adhesion molecule also known as CD15 (leucocyte cluster of differentiation 15) or SSEA-1 (stage-specific embryonic antigen 1) [14], [15]. Uchida *et al.* successfully isolated neural precursor cells from human foetal tissue using an antibody directed towards CD133 (Prominin) [16]. CD133 immunopurification was later applied by Lee *et al.* and Corti *et al.* to isolate neurosphere forming precursor cells from mouse foetal cerebellum and forebrain [17], [18]. In each of these cases, enriched cells expressed neural stem cell markers and were capable of multi-lineage differentiation both *in vitro* and *in vivo* [16]–[18]. Interestingly, CD133 expression could also be detected in a relatively small subpopulation of cells in brain tumours [19]. When these CD133+ cells were isolated, they were able to proliferate, form clonal neurospheres, and produce new tumors after serial transplantation [20]–[22]. CD133 positive cells are therefore considered as candidate cancer stem cells that maintain tumors [23]. Nonetheless, it is not clear whether all neural stem cells or brain cancer stem cells express CD133. Purified CD133+ or CD15+ cells become heterogeneous in neurosphere cultures, but this has been ascribed to differentiation [14], [16]–[18]. However, recent observations indicate that CD133 may not be present on the majority of slow dividing SVZ type B cells[24], considered as adult neural stem cells. Furthermore, tumorigenic cell lines have been derived from CD133 negative glioblastoma cells [25].

We previously reported the establishment of adherent mouse and human neural stem (NS) cell lines that are capable of clonal expansion and tri-potent differentiation [26], [27]. Unlike neurosphere cultures, NS cell lines are purified undifferentiated neural stem cell populations and therefore allow direct investigation of the stem cells. In this manuscript, we examine CD133 and CD15 expression in NS cell lines and the association with potency and cell cycle phase.

## Results

### Human NS cells exhibit heterogeneous CD133 and CD15 expression

**Figure 1:**
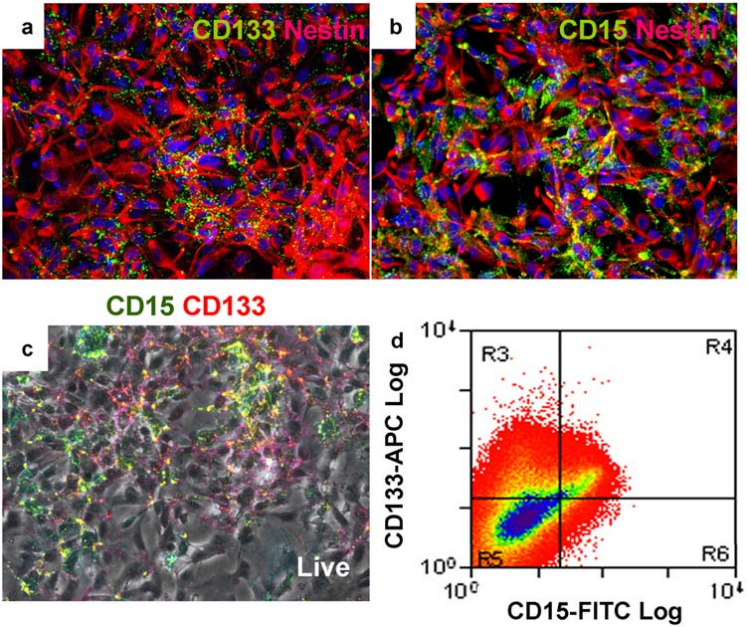
Human NS cells express CD133 and CD15 heterogeneously. Human NS cells homogeneously express the neural precursor marker Nestin (A and B, red), but exhibit heterogeneous CD133 (A, green) and CD15 (A, green) expression. Live cell staining (C) and subsequent flow cytometry analysis (D) reveal four sub-populations of human NS cells: CD133+/CD15^−^, CD133+/CD15+, CD133^−^/CD15+, and CD133^−^/CD15^−^. The proportions of each cell population are listed in Table 1.

To determine whether CD133 and CD15 are expressed by human neural stem cells in culture, we performed immunostaining on three independent human NS cell lines, CB541, CB660, and CB660sp [26]. CB541 and CB660 cell lines were derived from human foetal brain, and CB660sp was derived from human foetal spinal cord. All cell lines are maintained in monolayer culture conditions described previously [26]. In the presence of epidermal growth factor (EGF) and fibroblast growth factor 2 (FGF2), these cells remain undifferentiated and express a range of neural precursor and radial glia markers including Nestin, Sox2, Pax6, Vimentin, brain lipid binding protein (BLBP) and 3CB2 (Figure 1A and 1B, and data not shown). These markers are expressed relatively homogeneously throughout the NS cell population. In contrast, immunostaining of live or fixed cells reveals that human NS cells display heterogeneous expression of both CD133 and CD15 (Figure 1A–1C). Flow cytometry analysis confirmed this heterogeneity and showed that, on average, 31±2% of human NS cells express CD133 (CD133+/CD15±) and 10±2% express CD15 (CD133±/CD15+). The majority (68±2%) of cells do not express either marker, while 8±2% express both (Figure 1D and Table 1).

**Table 1:** Human NS cell lines display heterogeneous CD133 and CD15 expression (%)

	CD133+	CD133+	CD133^−^	CD133^−^
	CD15^−^	CD15+	CD15+	CD15^−^
CB541	21.73	11.03	1.67	65.57
CB660	21.94	7.27	2.46	68.33
CB660SP	22.47	7.18	1.20	69.15
Average	22.05±0.38	8.49±2.20	1.78±0.64	67.68±1.88

n = 750,000

**Figure 2:**
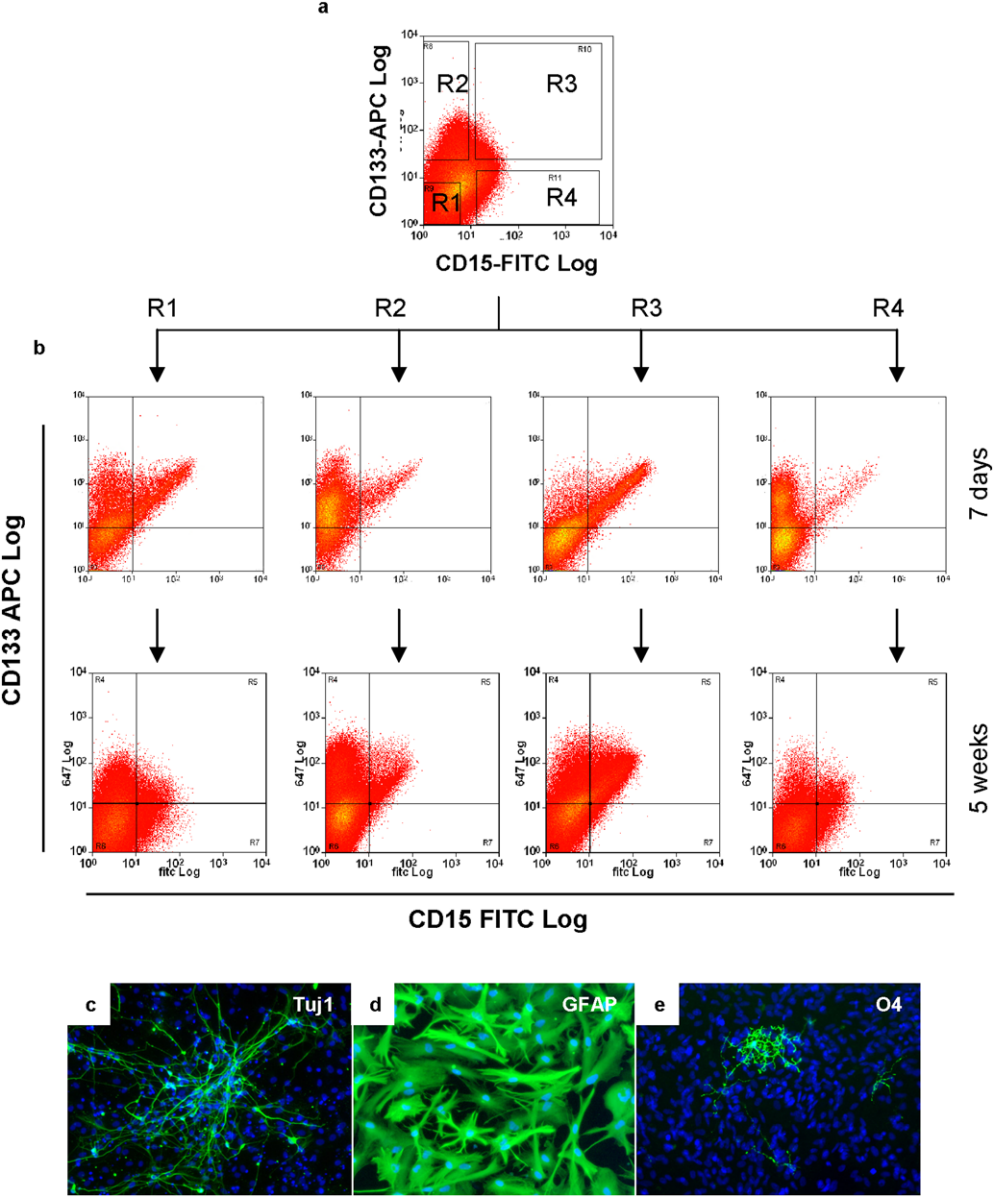
CD133 and CD15 expression varies in human NS cells but is not linked to stem cell potency. Human NS cells were flow sorted into four populations using the gates illustrated in (A) and then cultured in NS cell expansion conditions. One week later, each sorted cell population exhibited heterogeneous CD133 and CD15 expression (B). Four weeks later, all four cell populations displayed near-indistinguishable CD133 and CD15 distributions (B). Clonal cultures could also be derived from all four purified cell populations after cell sorting. The cloned cells retained tripotent, being able to generate neurons (Tuj1+) (C), astrocytes (high level GFAP with flattened morphology) (D), and oligodendrocytes (O4+) (E).

To investigate whether the variation in CD133 and CD15 expression might represent different cell populations in culture, we fractionated NS cells into CD133^−^/CD15^−^, CD133+/CD15^−^, CD133+/CD15+, and CD133^−^/CD15+ populations by flow cytometry using the gates illustrated in Figure 2A (purity ≥95%). Sorted cells were then cultured in self-renewal conditions in the presence of EGF and FGF2. Each population proliferated and after one week, we found all had re-acquired heterogeneous CD133 and CD15 expression (Figure 2B). When cultures were expanded for another 4 weeks, all four cell populations displayed similar CD133 and CD15 distribution (Figure 2B). In order to verify that CD133 and CD15 expression can switch within cell populations, we performed clonal analysis by depositing single human NS cells from each purified cell fraction. Colonies could be generated from single cells from all four NS cell populations. Immunostaining after 6 weeks expansion shows all NS cell clones exhibit heterogeneous CD133 and CD15 expression (data not shown). When further cultured, these colonies could generate clonal cell lines that were able to produce all three CNS cell types under appropriate differentiation conditions (Figure 2C–2E). The percentages of immunopositive neurons, oligodendrocytes, and astrocytes generated were 40–45%, 1–2%, and over 98% respectively. These values were similar between clones and in the same range as our previous report [26].

The above findings establish that clonogenic human NS cells do not express CD133 or CD15 constitutively. However, we noticed that colony formation efficiency did vary in relation to cell surface marker expression (Table 2). Surprisingly, the lowest frequency was from the double positive CD133+/CD15+ cells, only 3 out of 192 of which generated colonies. From 192 single CD133+/CD15^−^ and CD133^−^/CD15+ cells, 8 and 4 cells respectively produced colonies. Unexpectedly, the highest efficiency of 15% was from the CD133^−^/CD15^−^ population. These colonies were morphologically undifferentiated and indistinguishable from the parental human NS cell cultures.

**Table 2:** Colony formation efficiency of single deposited human NS cells

CD133+	CD133+	CD133^−^	CD133^−^
CD15^−^	CD15+	CD15+	CD15^−^
4.2%	1.6%	2.1%	15.0%

n =  192

### CD133 expression is regulated at the mRNA level in human NS cells

**Figure 3:**
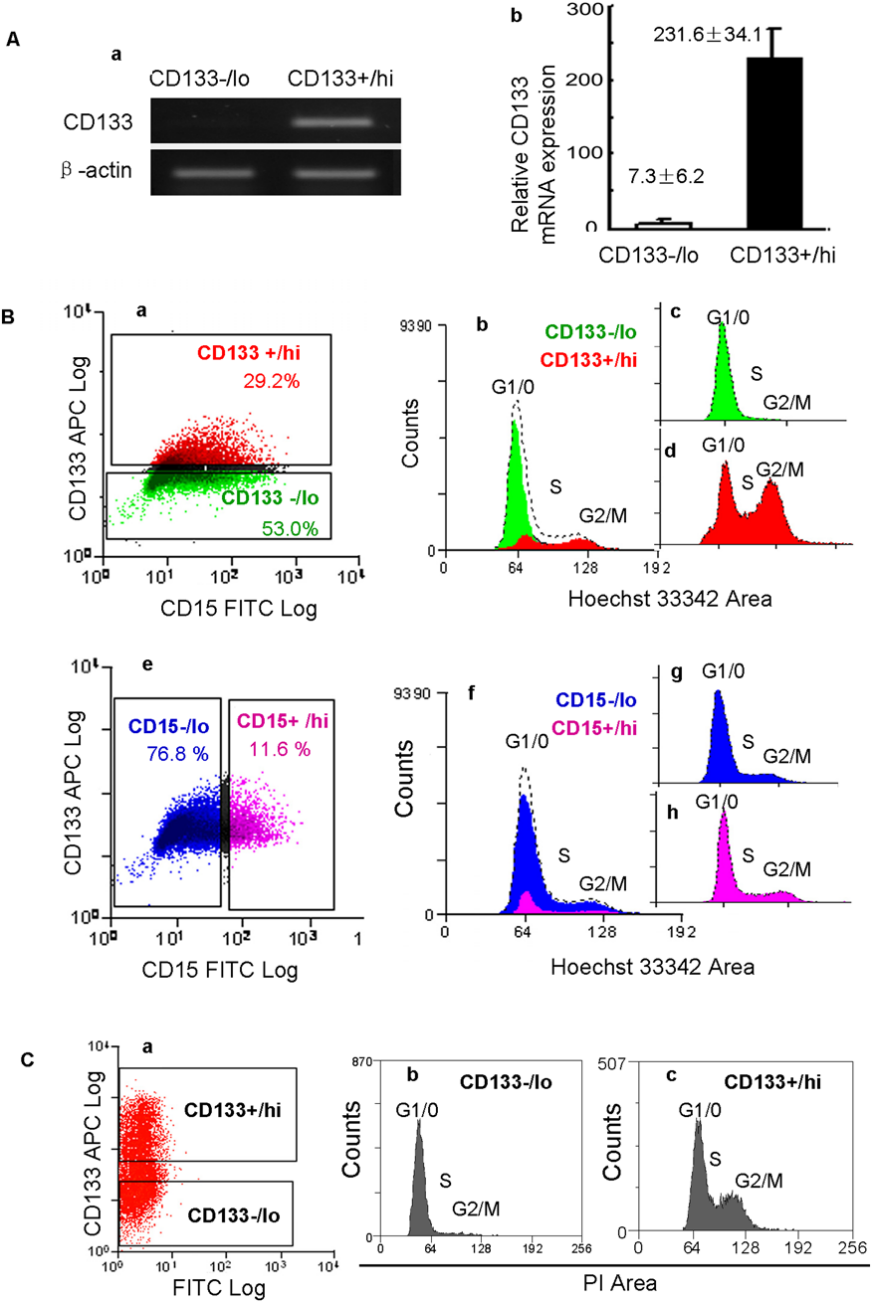
CD133 expression is regulated at the mRNA level and is reduced in G0/G1 phase. (A) RT-PCR (Aa) and real-time PCR (Ab) indicate that the expression of CD133 mRNA in CD133^−/lo^ human NS cells is approximately 30 fold lower in CD133^+/hi^ cells. CD133^−/lo^ and CD133^+/hi^ NS cells were purified by cell sorting using the gate illustrated in Ba below. (B) Cell cycle analysis of human NS cells stained with Hoechst 33342, anti-CD133-APC, and anti-CD15-FITC. Analysis gates were set as illustrated in Ba and Be. The CD133^+/hi^, CD133^−/lo^, CD15^+/hi^, and CD15^−/lo^ cells are colored red, green, pink, and blue respectively (Ba-h). Ungated cells are colored black. The cell cycle profile of the entire population is illustrated by dashed lines in Bb and Bf. The majority of CD133^−/lo^ (green) cells were in G1/G0 phase of the cell cycle (Bb and Bc), whereas over half of the CD133^+/hi^ (red) cells were in S, G2, or M phase (Bb and Bd). Although human NS cells express CD15 heterogeneously (Be), the CD15+^/hi^ (pink) and CD15^−/lo^ (blue) cell populations exhibited similar cell cycle profiles, consistent with the whole cell population (Bf-Bh). (C) To test the indication that CD133 may be preferentially down-regulated in cells in G0/G1 phase, we purified CD133^+/hi^ and CD133^−/lo^ cells using the gates illustrated in Ca and applied PI staining after fixation. Flow cytometry analysis indicated that the majority of CD133^−/lo^ cells were in G1/G0 (Cb), whereas over half of CD133^+/hi^ cells had >2N DNA content (Cc).

The above data suggest that CD133 and CD15 expression varies in human NS cell cultures and absence of these markers does not reflect loss of stem cell potency. To investigate whether CD133 expression in NS cells is regulated at the level of transcription or protein shedding [28], we collected mRNA from purified CD133^+/hi^ and CD133^−/lo^ human NS cells. RT-PCR and real-time PCR indicate that prominin/CD133 mRNA expression is down-regulated approximately 30 fold in CD133^−/lo^ cells compared to CD133^+/hi^ cells (Figure 3Aa and 3Ab).

Since the expression of some cell surface markers is related to cell density or cell cycle phase [29], we investigated whether CD133 and CD15 expression may be regulated by these factors. We first investigated human NS cells cultured at different densities. We plated human NS cells at 4×10^3^, 4×10^4^, and 4×10^5^ cells per cm^2^ respectively and expanded them in the same medium. Immunostaining and flow cytometry analysis performed 2 days later indicated that cells plated at low density exhibited similar heterogeneous distribution of CD133 and CD15 as high density cultures (Table 3).

**Table 3:** Human NS cells plated at different density exhibit similar CD133 and CD15 distribution (%)

	CD133+	CD133+	CD133^−^	CD133^−^
	CD15^−^	CD15+	CD15+	CD15^−^
4×10^3^ cells/cm^2^	19.71	10.04	1.89	68.36
4×10^4^ cells/cm^2^	19.09	8.23	2.56	70.12
4×10^5^ cells/cm^2^	21.37	6.19	1.19	71.25

n = 200,000

We next examined cell cycle correlation using Hoechst staining [30]. Based on 1.7×10^5^ scored events, flow cytometry analysis indicates that 65% of human NS cells are in G1/G0, 18% in S phase, and 17% in G2/M phase. We found 91% of CD133^−/lo^ cells have 2N DNA content, constituting 75% of all scored G1/G0 cells (Figure 3Ba–3Bd). Only 31% of CD133^+/hi^ cells were in G1/G0, while 69% were in S, G2, or M (Figure 3Ba–3Bd). In contrast, both CD15^+/hi^ and CD15^−/lo^ NS cells are distributed evenly throughout the cell cycle (Figure 3Be–3Bh). To test the indication that CD133 may be preferentially down-regulated in cells in G0/G1 phase, we purified CD133^+/hi^ and CD133^−/lo^ cells (purity≥95% and 98% respectively) (Figure 3Ca) and applied propidium iodide (PI) staining after fixation [31], [32]. Flow cytometry analysis indicated that over 88% of CD133^−/lo^ cells were in G1/G0, whereas approximately 65% of CD133^+/hi^ cells had >2N DNA content (Figure 3Cb and 3Cc).

### CD133 is also expressed heterogeneously by mouse NS cells

**Figure 4:**
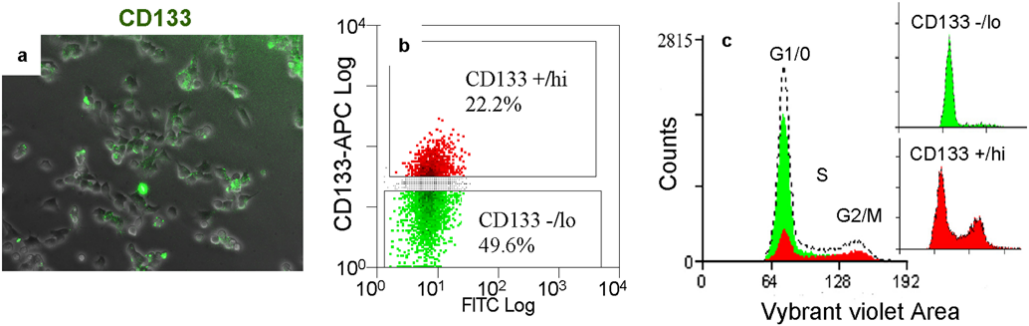
Mouse NS cells express CD133 heterogeneously. Live mouse NS cells exhibit heterogeneous CD133 expression (A). Flow cytometry indicated that 50% of CD133^−/lo^ cells (green) were in G1/G0 phase, whereas over half (51%) of the CD133+^/hi^ cells (red) were in S, G2, or M phase (B, C).

To investigate whether other mammalian neural stem cells express CD133 in a similar pattern, we applied similar staining and flow cytometry analysis to mouse NS cells [27]. Mouse NS cell lines derived either from embryonic stem (ES) cells (CGR8-NS cell line) or foetal cortex (Cor-1 cell line) exhibit heterogeneous CD133 expression (Figure 4A). On average, approximately 46±1% of mouse NS cells were immunopositive for CD133, and 54±1% of cells negative. Since Hoechst33342 shows considerable toxicity towards mouse NS cells, we applied an alternative dye, Vybrant® DyeCycle^TM^ Violet, to analyze cell cycle. Based on 5×10^5^ scored events, flow cytometry analysis indicated that 59% of mouse NS cells reside in G1/G0, 23% in S phase, and 18% in G2/M phase. Similar to observations in human NS cell cultures, CD133^−/lo^ cells were mostly 2N, while just over half (51%) of the CD133^+/hi^ cells have greater than 2N DNA content (Figure 4B and 4C).

### Human NS cell cultures may harbour slow-cycling or dormant stem cells

**Figure 5:**
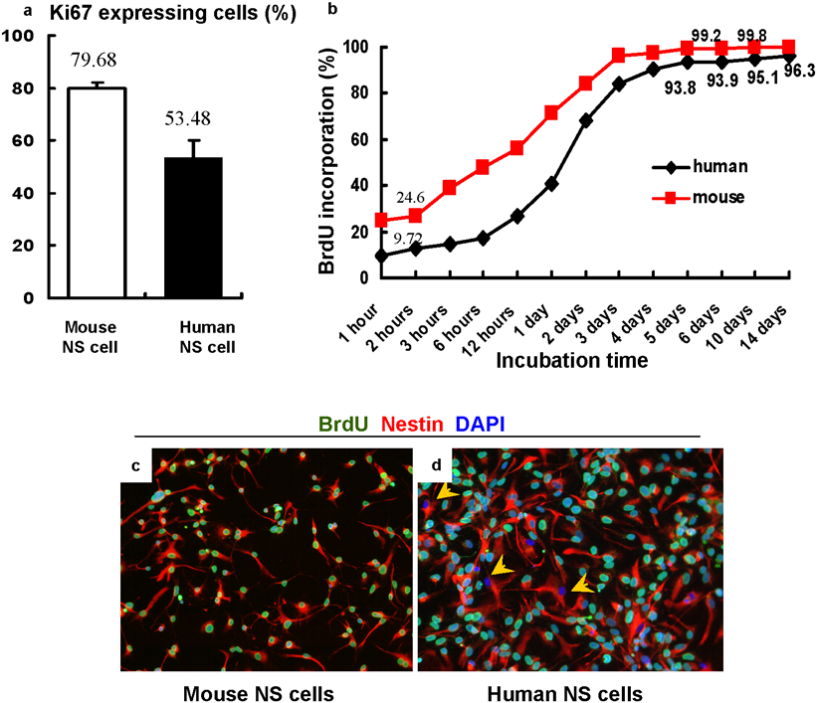
Human NS cell cultures propagate more slowly than mouse counterparts. Compared to mouse NS cell cultures, human NS cell cultures exhibit a lower percentage of Ki67 expressing cells under the same expansion conditions (A). Over 99% of mouse NS cells incorporated BrdU after 5 days incubation (B, C). Approximately 5% of human NS cells remained BrdU negative after prolonged incubation (B, D). Yellow arrows in Figure 5D indicate BrdU negative human NS cells after 10 days incubation.

The doubling time of human NS cell cultures (2–3 days) is much longer than that of mouse NS cells (∼24 hours) [26], [27]. Human NS cells also exhibit a higher percentage of CD133 negative cells that are mostly in G1/G0. Since human NS cell cultures do not express differentiation markers under expansion conditions [26], the longer doubling time is unlikely to be due to NS cell differentiation into non-proliferative cell lineages. We speculated that a fraction of human NS cells may spontaneously withdraw from the cell cycle (entering G0) which could contribute to a prolonged doubling time. We performed immunostaining against Ki67, a nuclear protein expressed by proliferating cells in all cell cycle phases. We found 79.68±2.40% of mouse NS cells expressed Ki67, but only 53.48±6.62% of human NS cells exhibited Ki67 immunoreactivity under identical culture conditions (Figure 5A). Bromo-deoxyuridine (BrdU) incorporation experiments revealed that mouse NS cell cultures contained a higher percentage of BrdU labelled cells at all checked time points, reaching a plateau close to 100% after ∼3 days incubation (Figure 5B and 5C). In contrast, the fraction of BrdU+ human NS cells began to level off at ∼95% after 4 days incubation (Figure 5B and 5D). This suggests that up to 5% of human NS cells may be slow-dividing or non-dividing, although they have not differentiated into neurons or glia.

**Figure 6:**
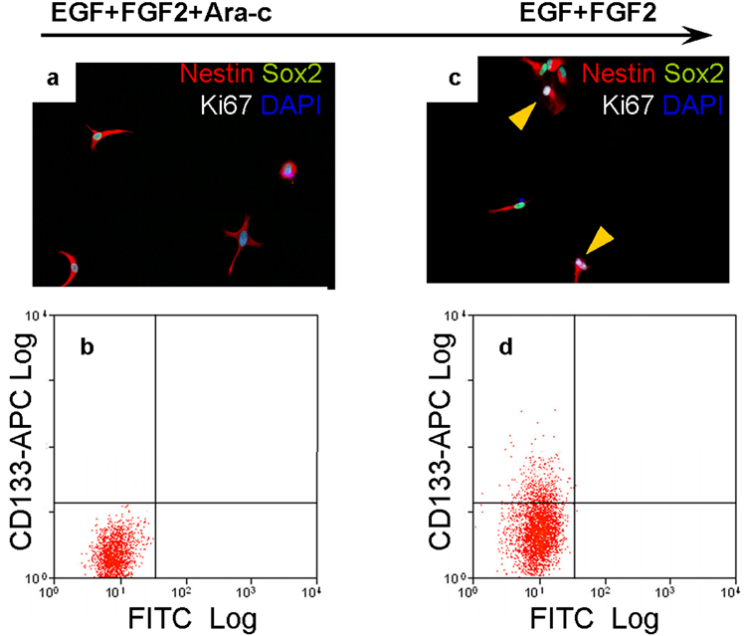
Human NS cell cultures harbour slow-cycling or dormant cells. Approximately 5% of human NS cells remained viable after 5 days exposure to the antimitotic drug Ara-c. The viable cells retained Nestin and Sox2 expression but did not express Ki67 or CD133 (A, B). When these cells were re-plated into medium without Ara-c for 10 days, approximately 14.6% of cells expressed Ki67 and 8.7% expressed CD133 (C, D). Yellow arrows in (C) indicate Ki67 positive cells.

To test the hypothesis that human NS cell cultures may harbour dormant or slow-dividing cells, we attempted double staining with Hoechst and Pyronin Y [33], [34]. However, this combination proved extremely toxic and over 99% of human and mouse NS cells died after staining. We therefore applied the antimitotic drug cytosine-β-d-arabinofuranoside (Ara-c, 2%) [35], [36] to eliminate dividing human NS cells. Approximately 5% of human NS cells remained viable after 5 days Ara-c treatment. Immunostaining showed these cells retained Nestin and Sox2 expression, but they were not positive for CD133, Ki67, or Caspase-3 (Figure 6A and 6B, and data not shown). Upon re-plating without Ara-c and culturing for 10 days in EGF and FGF, approximately 15% of cells displayed Ki67 expression and 8.7% expressed CD133 (Figure 6C and 6D). When further cultured, these cells expanded continuously and could differentiate as other NS cells (data not shown).

## Discussion

Cell surface markers CD133 and CD15 have been recognized as neural stem cell markers and have been applied to enrich for neural precursor cells from various sources [14], [16]–[18], [37]. However, to our knowledge, purified CD133 or CD15 expressing neural precursor cells have not been maintained as homogeneous cell populations *in vitro*. In neurosphere cultures this is obscured because neural precursor cells spontaneously differentiate into glial lineages [38]–[40]. By analyzing purified human NS cell lines [26], we find CD133 and CD15 are only expressed by a subpopulation of neural stem cells. We demonstrated that single CD133 and/or CD15 negative cells could generate clonal and tripotent neural stem cell lines. The two cell surface markers were invariably expressed heterogeneously in proliferating cultures. Therefore cultured neural stem cells are not constitutively CD133 or CD15 positive. This could be related to the observation that CD133 expression appears linked to cell cycle phase in NS cells. CD133 negative cells are disproportionately represented in G0/G1. Recently, cell cycle dependent variation in CD133 expression has been described for colon cancer and melanoma cell lines [41]. These authors also find that CD133 levels are down-regulated in G0-G1, suggesting that this may be a generic feature.

In our adherent culture conditions CD133 negative human NS cells exhibited higher colony formation efficiency than CD133 positive cells. Previous analyses using primary human foetal CNS tissue have indicated that few or even no neurospheres could be derived from CD133 negative cell populations [16]. However, primary tissues contain overwhelming numbers of differentiated cells that are CD133 negative. Therefore CD133 sorting will give a major enrichment even if only capturing a fraction of the stem cells. It is also possible that CD133 negative stem cells may not initiate colony formation in suspension culture, even though they do so efficiently when adherent. The higher cloning efficiency we observed for CD133 negative versus CD133 positive cells might be because G0/G1 cells are intrinsically more clonogenic, or they may be less fragile and more resistant to flow sorting compared cells in S, G2, or M phase. Alternatively they may have some other feature that resists stress-induced differentiation or promotes attachment.

Interestingly, although human and mouse NS cells are cultured under identical conditions, human NS cell cultures harbour fewer CD133+ cells and exhibit a longer doubling time than mouse counterparts. Ki67 staining and BrdU incorporation experiments indicated that a subpopulation of human NS cells may withdraw from the cell cycle. The fraction of non-cycling human NS cells, estimated from BrdU incorporation (Figure 5B) is around 5% whereas this is less than 0.2% in mouse NS cell cultures. These non-cycling cells do not show features of differentiation or senescence, and retain precursor markers suggesting they could be dormant stem cells. To investigate further we applied Ara-c, an antimitotic drug, to eliminate dividing cells. Approximately 5% of human NS cells remained viable. These cells retained Nestin and Sox2 expression but did not express CD133 or Ki67. Parallel BrdU labelling confirmed they were not dividing. Crucially however, a proportion of these cells resumed proliferation after the antimitotic drug was removed. This strongly suggests that human NS cells in culture can suspend proliferation but retain the capacity to re-enter the cell cycle.

A major interest in neural stem cell biology is the relationship with brain tumor cells [42,43 [44]]. CD133 has been successfully applied to isolate brain tumor initiating cells, also called cancer stem cells [19], [20]. It was found that malignant brain tumors have a higher CD133 index than low-grade tumors [19]. Based on the present observations, however, this does not necessarily indicate an increased frequency of stem cells but may reflect a higher proliferative index in the stem cell compartment. Indeed we have found that glioblastoma stem cells also exhibit heterogeneous CD133 and CD15 expression *in vitro* [44], similar to the profile observed in human NS cells. If CD133 expression in brain cancer stem cells is regulated in a cell cycle dependent fashion, extra caution must be taken when this marker is used to define tumour stem cells. This would be particularly significant if CD133 is not expressed by quiescent brain cancer stem cells.

In conclusion, our data demonstrate that mammalian neural stem cells are not constitutively CD133 or CD15 positive, and that down-regulation of CD133 protein and mRNA correlates with an enrichment of cells in G0-G1 phase of the cell cycle. These observations point to the potential absence of CD133 expression in slow-cycling or dormant neural stem cells. It will be informative to investigate whether CD133 expression is similarly down-regulated in G0/G1 in adult neural stem cells *in vivo*. The mechanisms and regulation of NS cell dormancy are also of interest for future study.

## Methods

### Cell culture

The derivation of human and mouse NS cell lines is described in [26] and [27]. Established human and mouse NS cell lines are cultured on laminin (10 mg/L, Sigma) coated dishes (Iwaki) in expansion medium comprising RHB-A medium (Stem Cell Sciences Ltd., UK), recombinant mouse EGF (10 ng/ml, Peprotech), and recombinant human FGF-2 (10 ng/ml, Peprotech). Expansion medium was changed every 2 days. Cells were detached and split 1∶2 to 1∶3 using Accutase (Sigma) once cultures became confluent. Clonal NS cell lines were generated by deposition of single cells into laminin coated 96-well plates using a Dako Cytomation MoFlo cell sorter. The presence of single cells was checked under a bright field microscope after sorting. Culture medium was renewed by 50% change every 3 days for clonal expansion. Clones were passaged after 4 weeks. Differentiation protocols for generating neurons, astrocytes, and oligodendrocytes from NS cells are described in [26] and [27].

### Immunostaining

For intracellular staining, cells were fixed with 4% PFA for 15 minutes at room temperature followed by 30 minutes incubation with BLOCK solution. Each 100 ml BLOCK solution contained 97 ml PBS, 3 ml serum, and 0.1% Triton-X100. Cells were then incubated with primary antibodies for 2 hours at room temperature or overnight at 4°C. We used Alexa-Fluor secondary conjugates (Invitrogen) and DAPI (Sigma) to visualize the staining. Primary antibodies were used at the following dilutions: Nestin (1∶500; R&D Systems), Sox2 (1∶400; Chemicon), GFAP (1∶300; Millipore), Tuj1 (1∶200, Covance), 3CB2 (1∶20, DSHB), BLBP (1∶500, Abcam), Vimentin (1∶20, DSHB), and Ki67 (1∶250, Lab Vision).

To stain live cells, cultured cells were incubated with primary antibody in serum-free expansion medium for 15 minutes at room temperatures followed by 3 washes with medium. Staining was visualized by incubation with Alexa-Fluor secondary conjugates (Invitrogen) for 10 minutes at room temperatures. Primary antibodies were used at the following dilutions: anti-human CD133 (1∶10, Miltenyi Biotec), anti-mouse CD133 (1∶5, eBioscience), anti-human CD15 (1∶10, Miltenyi Biotec), and anti-O4 (1∶100, R&D Systems). Live stained cells could subsequently be fixed and stained with further antibodies.

For BrdU assays, NS cells were plated into 12-well plates with expansion medium and were incubated at 37°C overnight for recovery. Cells were then expanded in the presence of 10 µM BrdU (Sigma). Cells were fixed in 4% paraformaldehyde at different time points. After fixation, we added 500 µl of 2M HCl into each well and left at room temperature for one hour. Cells were then stained with anti-BrdU antibody (Sigma) using protocols described above.

### Semi-quantitative and real-time RT–PCR

We used RNeasy kit (Qiagen) to extract total RNA and Superscript III (Invitrogen) to prepare cDNA. cDNA concentrations were determined and normalized by NanoDrop-1000 (Thermo Scientific). RT-PCR was performed for 30 cycles for all markers except β-actin for 25 cycles (denaturing for 40 s at 94°C; annealing for 40 s at 56°C, extension for 60 s at 72°C). PCR products were resolved on 1.5% agarose gel. Real-time PCR was carried out using a LightCycler (Roche). Primers were designed using MIT Primer3 software as follows: β-actin forward primer 5′-GTC TTC CCC TCC ATC GTG-3′, β-actin reverse primer 5′-AGG TGT GGT GCC AGA TTT TC-3′, CD133 forward primer 5′-CAG AGT ACA ACG CCA AAC CA-3′, CD133 reverse primer 5′-AAA TCA CGA TGA GGG TCA GC-3′.

### Flow cytometry and cell sorting

Single-cell suspensions were stained with antibodies against human CD133 (1∶10, APC conjugated, Miltenyi Biotec), mouse CD133 (1∶5, APC conjugated, eBioscience), and human CD15 (1∶10, FITC conjugated, Miltenyi Biotec). Dead cells were excluded using forward and side scatter as well as the vital dye Topro-3 (Molecular Probes) or 7AAD (BD). Typically 1 million cells were stained in an eppendorf tube. Antibodies were added into serum-free medium and incubated in the dark for 15 minutes at room temperatures. Cells were then centrifuged and washed 3 times in medium prior to analysis or sorting. Flow cytometry analysis was carried out on a CyAn ADP analyzer (Beckman). Cell sorting was performed using a MoFlo sorter (Dako Cytomation). Matched isotype antibodies were applied in parallel as controls.

Cell cycle was analyzed using two methods. For live NS cells, a single cell suspension containing approximately 1 million cells was incubated with 0.5 µg Hoechst 33342 (Invitrogen) or 0.5 µg Vybrant DyeCycle Violet (Invitrogen) at 37°C for 30 minutes in 1 ml medium. Alternatively, the cell suspension was fixed in 70% ethanol at −20°C for 15 minutes. Cells were then centrifuged and incubated in 5 ml PBS at room temperature for 15 minutes. Finally, approximately 1 million cells were transferred to PBS containing 0.5 mg/ml propidium iodide (Invitrogen) and 1 mg/ml RNAse (Sigma). Cell cycle analysis was carried out on a CyAn ADP analyzer (Beckman) and using Flowjo software (Tree Star, Inc). CD133/CD15 staining was performed on live cells before Hoechst and propidium iodide staining in cases of triple staining.
